# POPPIE: protocol for a randomised controlled pilot trial of continuity of midwifery care for women at increased risk of preterm birth

**DOI:** 10.1186/s13063-019-3352-1

**Published:** 2019-05-14

**Authors:** C. Fernandez Turienzo, D. Bick, M. Bollard, L. Brigante, A. Briley, K. Coxon, P. Cross, A. Healey, M. Mehta, A. Melaugh, J. Moulla, P. T. Seed, A. H. Shennan, C. Singh, R. M. Tribe, J. Sandall

**Affiliations:** 1 Department of Women and Children’s Health, King’s College London, St Thomas’ Hospital, London, SE1 7EH UK; 2 Warwick Clinical Trials Unit, Warwick Medical School, University of Warwick, Gibbet Hill, Coventry, CV4 7A UK; 3 Lewisham and Greenwich NHS Trust, Lewisham High Street, London, SE13 6HL UK; 4 Florence Nightingale Faculty of Nursing, Midwifery and Palliative Care, King’s College London, James Clerk Maxwell Building, London, SE1 8WA UK; 5 Department of Midwifery, Kingston University and St. George’s, University of London, Hunter Wing, Cranmer Terrace, London, SW17 0RE UK; 6 Department of Public Health, London Borough of Lewisham, Laurence House, London, SE6 4RU UK; 7 Centre for Implementation Science, Institute of Psychiatry, Psychology and Neuroscience, King’s College London, David Goldberg Centre, De Crespigny Park, Denmark Hill, London, SE5 8AF UK

**Keywords:** Preterm birth, Maternity care, Maternal health services, Models of care, Continuity of care, Hybrid type 2 implementation/effectiveness trial, MRC guidance complex interventions

## Abstract

**Background:**

High rates of preterm births remain a UK public health concern. Preterm birth is a major determinant of adverse infant and longer-term outcomes, including survival, quality of life, psychosocial effects on the family and health care costs. We aim to test whether a model of care combining continuity of midwife care with rapid referral to a specialist obstetric clinic throughout pregnancy, intrapartum and the postpartum period is feasible and improves experience and outcomes for women at increased risk of preterm birth.

**Methods:**

This pilot, hybrid, type 2 randomised controlled implementation trial will recruit 350 pregnant women at increased risk of preterm birth to a midwifery continuity of care intervention or standard care. The intervention will be provided from recruitment (antenatal), labour, birth and the postnatal period, in hospital and community settings and in collaboration with specialist obstetric clinic care, when required. Standard care will be the current maternity care provision by NHS midwives and obstetricians at the study site. Participants will be followed up until 6–8 weeks postpartum. The composite primary outcome is the appropriate initiation of any specified interventions related to the prevention and/or management of preterm labour and birth. Secondary outcomes are related to: recruitment and attrition rates; implementation; acceptability to women, health care professionals and stakeholders; health in pregnancy and other complications; intrapartum outcomes; maternal and neonatal postnatal outcomes; psycho-social health; quality of care; women’s experiences and health economic analysis. The trial has 80% power to detect a 15% increase in the rate of appropriate interventions (40 to 55%). The analysis will be by ‘intention to treat’ analysis.

**Discussion:**

Little is known about the underlying reasons why and how models of midwifery continuity of care are associated with fewer preterm births, better maternal and infant outcomes and more positive experiences; nor how these models of care can be implemented successfully in the health services. This will be the first study to provide direct evidence regarding the effectiveness, implementation and evaluation of a midwifery continuity of care model and rapid access to specialist obstetric services for women at increased risk of preterm birth.

**Trial registration:**

ISRCTN37733900. Retrospectively registered on 21 August 2017.

## Background

Preterm birth (PTB) is a birth that occurs at < 37 weeks of gestation. PTB rates are increasing worldwide, despite advances in antenatal care [1]. The cost of PTB to the UK National Health Service (NHS) is estimated to exceed £3 billion per annum [2]. PTB is associated with poorer quality of life [3], with no single strategy effective in reducing rates [4]. There are multiple known risk factors associated with early birth including previous PTB, late miscarriage and cervical surgery, but the cause of spontaneous PTB is unknown in approximately 40–50% of cases [5].

A Cochrane review of 15 trials involving 17,674 women reported that women randomised to receive continuity of care by one named midwife, or a small group of midwives, during pregnancy, birth and the postnatal period were significantly more likely to be attended at birth by a known midwife. They were significantly less likely to experience PTB or fetal loss/neonatal death before 24 weeks and had fewer fetal/neonatal deaths in total [6]. There is also evidence of increased referral to specialist services and clinical benefit for women with social complexity [7].

Screening and managing women at increased risk of PTB in specialist obstetric clinics is now considered useful [8]. However, PTB clinics or preterm surveillance clinics are rarely available outside research settings, and women who require specialist medical and obstetric expertise often receive fragmented midwifery care. Continuity of carer is identified in the current maternity policy document ‘Better Births’ as a model of care that the NHS England is committed to increase. However, around 75% of women have not met the midwife who attends their birth beforehand [9]. Our research [10–12] provides a basis to develop and test the impact of a novel care pathway for women at increased risk of PTB, which combines continuity of midwife care with rapid referral to a specialist obstetric clinic throughout pregnancy through to the intrapartum and postpartum periods [13].

POPPIE (Pilot study Of midwifery Practice in Preterm birth Including women’s Experiences) is a pilot, hybrid, type 2 randomised controlled implementation trial to determine whether this model of continuity of care is feasible, and could improve pregnancy outcomes and women’s experiences. The setting will be an inner-city hospital in South London.

**Fig. 1:**
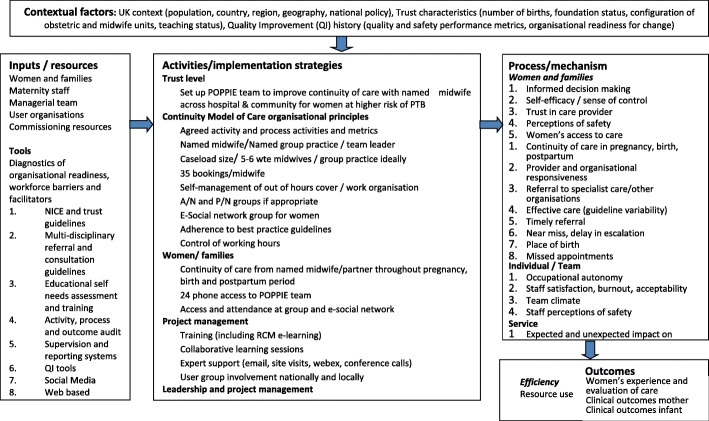
POPPIE logic model for research

The TIDieR guidance [14] will be used to describe the intervention and standard care. In order to clarify assumptions in relation to how the intervention will be implemented, and the mechanisms through which it will produce change in this context, a logic model informs data items for the implementation outcomes assessment [15] (Fig. 1). We will use the Reach Effectiveness Adoption Implementation Maintenance (RE-AIM) [16] and Medical Research Council (MRC) [17] guidance for the evaluation of complex interventions to assess feasibility, acceptability to staff and women, adoption, fidelity, reach, equity, penetration, costs and sustainability, and to inform implementation and the mechanisms of impact on maternal and neonatal health, women’s experiences of care and quality of care.

### How the intervention may work?

Continuity of care is defined as delivering care that acknowledges that a woman’s health needs are related to events and should be managed over time [6]. Therefore, midwifery continuity of care allows women to develop a relationship with the same caregivers throughout pregnancy, birth and the postnatal period. Women have a named and a ‘backup’ midwife, from the same team, caring for them during labour, birth and the postpartum period, whom they have met before and feel they know. This trusting relationship may increase their confidence and reduce levels of psychosocial stress [18, 19]. This close relationship between women and midwives may also help women feel that they have a health care provider who knows their medical history who will be caring for them throughout labour, birth and the postnatal period [20, 21]. The trust and feeling of safety that this may afford may mean that women are more willing to accept help from psychiatric services, domestic violence advocacy and other services, where required. Women may be more likely to talk about lifestyle choices, such as smoking and drinking, and to trust the advice they receive [7, 22]. However, women may also be unhappy with a perceived intrusive surveillance.

It is also hypothesised that there is an improved co-ordination and collaboration between midwives working in continuity models of care and the multi-disciplinary network of support, improved access to care, improved care according to guidelines, earlier recognition of problems and improved health behaviours. Thus, the initiation of treatments to prevent or reduce PTB may occur earlier in the intervention group because the midwives have greater knowledge, there are lower barriers for women to contact midwives and faster access to obstetric opinion and management.

## Methods

The aim of this study is to test in a pilot study if a model of care combining continuity of midwife care with rapid referral to a specialist obstetric clinic for women at increased risk of PTB is feasible and improves experience, pregnancy outcomes and quality of care. If feasibility is confirmed, learning from this pilot trial will inform a full-scale multi-centre trial, following the MRC guidelines [17] – aimed at improving outcomes and reducing the incidence and appropriate and timely management of PTB.

### Design, setting and population

**Fig. 2:**
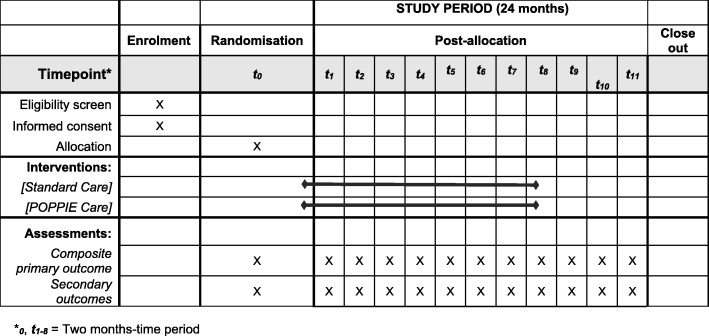
Standard Protocol Items: Recommendations for Interventional Trials (SPIRIT) adapted for the POPPIE trial

We will undertake a two-armed, parallel-design, individually pilot randomised controlled trial. A type 2 hybrid design will place equal focus on evaluating the effectiveness of the intervention and the implementation [23]. The study will be undertaken in an inner-city hospital in South London with a higher than national average PTB rate (8.2 vs 7.2% in England) [24]. The study population will include pregnant women booking for antenatal care, identified as at increased risk of PTB and residing in the hospital catchment area in the study period (Fig. 2).

### Participants

We will include asymptomatic pregnant women with singleton pregnancies attending for antenatal care at less than 24 weeks’ gestation and identified at increased risk of PTB – fulfilling one or more of the following criteria: previous cervical surgery (such as cone biopsy, loop diathermy); uterine abnormality (such as bicornuate uterus); previous short cervix or short cervix this pregnancy (< 25 mm); previous cerclage; previous premature ruptured membranes (< 37 weeks); one or more previous PTB (< 37 weeks); one or more previous late miscarriage/abortion (> 14 weeks); current smoker of tobacco, as identified at first antenatal appointment. We will exclude women aged < 18 years at recruitment, multiple pregnancies, unable/unwilling to give informed consent and those already receiving care from a specialist midwifery team (e.g. women with severe mental illness, diabetes, alcohol and substance misuse).

### Identification, screening, recruitment and randomisation

Trial information will be available in all relevant clinical areas including maternity services, general practice surgeries and health centres. Community midwives and other maternity care clinicians will refer women who express an interest in the study to the midwifery team leader to discuss trial participation. At early nuchal and/or anomaly ultrasound scan appointments, women who meet inclusion criteria will be offered verbal and written information about the study. Potentially eligible women will also be identified by review of medical records and appointment lists by clinical staff. In these cases, women will be contacted by a member of the direct clinical team to discuss their eligibility and study participation and sent a leaflet by post/email to read (if interested) before their appointment attendance. Adequate time will be given to interested women to explain the study, for considering participation, and asking any questions with members of the clinical team. All participants will provide written informed consent prior to participation.

**Fig. 3:**
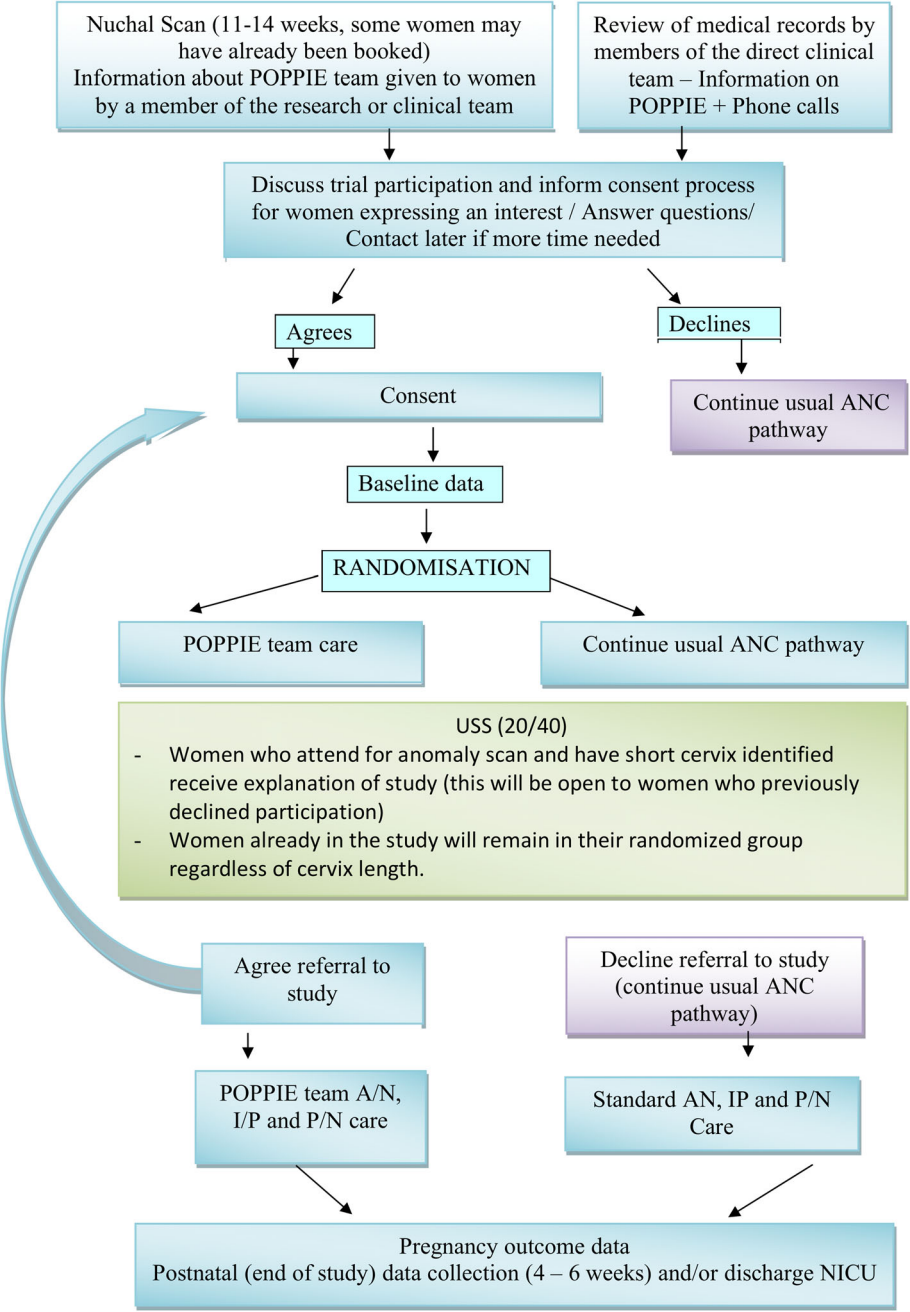
Flow diagram of participants

Baseline data will be collected, and participants randomised in a 1:1 ratio to receive either POPPIE care or routine standard care. Randomisation will be managed via a secure web-based randomisation system (MedSciNet™), which is part of a password-protected Internet-based study specific data management system. A minimisation algorithm will be used to ensure balance between the groups with respect to smoking at booking and previous PTB (< 37 weeks). The nature of the intervention is such that blinding of participants and care providers cannot be achieved. However, risk of bias will be reduced since data will be processed by researchers blinded to the model of care that the women is allocated to. The flow diagram of participants though the study is shown in Fig. 3.

### Intervention: POPPIE midwifery team

Women allocated to the intervention group will receive a continuity of midwife care model during the antenatal, labour, birth and postnatal periods, predominantly from one midwife and her partner midwife (backed up by the rest of the POPPIE team). Midwives will ‘follow women’ and scheduled and out of hours’ antenatal, intrapartum and postnatal care may be provided in hospital, community/children’s centres or at home. Midwives will provide continuity of care in a multi-disciplinary network of consultation and referral with other care providers. Some antenatal and/or intrapartum and/or postpartum care will be provided in consultation with medical staff, as appropriate [5]. Within this model, midwives work in partnership with the woman; they are the primary professional with responsibility for assessment of needs, planning care, referral to other professionals as appropriate, and for ensuring co-ordination of services. Clinical care content will follow NHS Trust hospital guidelines. The team will have rapid access using a mobile phone to a linked consultant obstetrician with specialist expertise in PTB to refer and/or discuss any clinical concerns, issues or queries.

The POPPIE team will include six Whole-time Equivalent (WTE) midwives, including an experienced senior midwife who leads the team. Specialist training (preventing and managing PTB; caring for preterm infants; bereavement; working in midwifery continuity of care models), research training (Good Clinical Practice); valid informed consent; data collection and data entry) and ongoing support will be provided following a training needs assessment.

### Control: Standard midwifery care

Women allocated to standard care will receive care as appropriate from the local maternity services in line with usual practice at the study site, across the antenatal, labour, birth and postnatal periods. Antenatal care is provided by different midwives working in the community, children’s centres, and/or hospital. Some antenatal and/or intrapartum and/or postpartum care is also provided in consultation with medical staff as appropriate. Core hospital midwives and/or physicians in the birth centres and/or labour ward provide labour and birth care and midwives in the postnatal ward provide postnatal care. Women are also offered midwifery visits at home and in postnatal clinics in the community following discharge from hospital by a range of midwives. Clinical care content follows Trust hospital guidelines. Midwives in the standard care arm do not have a linked obstetrician working directly with them and they may have to contact on-call obstetric registrars, obstetricians or other services (e.g. antenatal clinics, day assessment unit) to discuss any clinical concerns, issues or queries or make referrals.

### Intervention and control: Obstetric care pathway

In accordance with the hospital guidelines, women identified as being at increased risk of PTB in both trial arms are seen by a consultant obstetrician as soon as possible after their nuchal scan to discuss an individual care plan based on their obstetric and medical history. Women in both trial arms with specific risk factors for PTB are then followed up weekly or 2-weekly from 14 to 24 weeks’ gestation in the cervical scan clinic, as considered necessary. Depending on risk factors, women may be referred to a consultant obstetrician and/or offered additional tests (e.g. a transvaginal scan, a urine test and/or a vaginal swab, a fetal fibronectin test) and other preterm interventions according to Trust guidelines (e.g. cervical cerclage, antibiotics, progesterone, steroids) with follow-up appointments in the clinic up to 30 weeks of pregnancy.

### Outcomes and measures

#### Primary outcomes

The composite primary outcome is the initiation of any of the following interventions appropriately provided for the prevention and/or management of preterm labour and birth (antibiotics for urinary tract infections, smoking cessation and domestic violence advocacy referrals, transvaginal scan assessments of the cervix, fetal fibronectin assessments, cerclage insertion, progesterone administration, corticosteroid administration, magnesium sulphate administration, admission for observation and in utero transfer). A composite outcome is chosen, as the primary outcome as powering a pilot trial for PTB alone would require too large a sample size, and helps the investigators to avoid an arbitrary choice between several important outcomes that refer to the same disease process [25]. We will report the effect of the continuity of care model on both the composite and its components for all woman in the study, specifying if (1) an intervention was required and (2) the intervention was provided. Women who experience any one of the intervention components will be considered to have experienced the composite outcome.

#### Secondary outcomes

The secondary outcomes are related to recruitment and attrition rates; acceptability to women, health care professionals and stakeholders; health in pregnancy and other complications; labour and birth outcomes; maternal and neonatal postnatal outcomes; assessment of psycho-social health; quality of care and women’s experiences of trust, stress, system responsiveness and safety. Measures of implementation outcomes (acceptability, adoption, reach, fidelity, appropriateness, penetration, costs, resource use and sustainability) will be guided by the RE-AIM Framework [16].

### Data collection

Demography, medical history and current pregnancy health information and psycho-social health data will be collected prior to randomisation at baseline visit (recruitment). Follow-up data will continue up to 6–8 weeks postpartum for mothers and until discharge from neonatal intensive care unit (NICU) for babies (up to 3 months). Pregnancy outcome, birth and neonatal data will be collected from maternal and infant medical records and NHS electronic data systems. Data from validated questionnaires at baseline and postnatally (e.g. Prenatal Distress Questionnaire [26], Everyday Discrimination Scale [27], PHQ1 Mental Health [28], PROMIS-10 Health Related Quality of Life [28], Labour Agentry Scale [29], Mother-to-Infant Bonding Scale [28, 30], Trust in Nurses Scale [31], Social Support [32]) will be used to assess health and wellbeing, and measure women’s experiences, continuity, quality of care and safety. Data will be anonymised and entered onto the secure, password-protected MedSciNet™.

### Withdrawal

Participants can withdraw at any point without giving a reason. Women may be withdrawn from the trial if midwifery specialist care is required (e.g. mental health support). Permission will be sought to access routinely collected clinical pregnancy outcome data.

### Process and implementation evaluation

The process and implementation evaluation will aim to understand variations in the impact of the intervention on outcomes of interest and to contextualise findings. Activity data will be integrated with planned process evaluations using both quantitative and qualitative methods. We will interview 30 women in both intervention and control groups to assess acceptability and explore experience. Sampling for interviews will be based on maximum diversity in relation to age, socio-demographic characteristics, parity and obstetric history. Trial recruitment and retention will be assessed by examining eligibility, participation and attrition rates. Fidelity will be assessed through routine monthly audits and include access to care, completeness of antenatal care, content of the intervention, continuity of care (number of midwives seen), referrals. Up to 20 key stakeholders including midwives, clinicians, managers and commissioners will be interviewed regarding acceptability of working in a new model of care and the impact on the wider organisation.

### Data analysis

#### Sample size calculation

There is no pre-existing precise data for the composite outcome that we plan to use. However, we consider that a 15% increase in the rate of appropriate interventions is both feasible and clinically useful. With six WTE midwives in the POPPIE team, we can randomise 175–210 women to the active intervention (35 bookings per year per midwife) during a 12-month period. The standard care arm is limited by the total number of women available, which is likely to be less than 400 per year. With 175 women in each arm (350 in total), we would have 80% power to detect a 15% increase in the rate of appropriate interventions (from 40 to 55%) (calculated using the Stata version 14.2 (StataCorp, College Station, TX, USA)).

#### Proposed statistical analysis

The analysis will be ‘intention to treat’, including withdrawals and losses to follow-up. Randomisation should ensure that the groups are similar or equivalent in their baseline characteristics; additional multivariate analysis will be used if baseline differences are noted between the two groups.

The relative risks (with 95% confidence intervals) of the component primary outcomes will be calculated. Secondary outcome measures of categorical data will be analysed with *χ*^2^ tests and continuous data will be analysed with *t* tests (for normally distributed data). Ranked or Likert-scale data will be analysed using cumulative odds ratios. Logistic regression and multiple linear regressions will be used if necessary to adjust for any other confounding variables. Outcome assessments and analysis will be blinded.

#### Health economic analysis

For the primary analysis, the resource use data related to health care services utilised by the trial participants, such as antenatal, intrapartum, neonatal care and postnatal visits, hospital overnight stays and readmission, will be considered. These data will be collected for both intervention and control arm from clinical records and the postnatal survey.

Unit cost estimates will be applied to resource use data to generate individual-level cost estimates. Sources of unit costs will include routine or published literature such as the latest NHS Reference Costs. In addition, published sources and information from local health care providers will be sought. For the non-health related resources, unit costs will be sought from the relevant sources such as governmental websites. Standard statistical methods for analysing trial-based economic data will be used to evaluate the resource and cost-impacts of the continuity of care model compared to standard practice. We will adopt a ‘cost-consequences’ approach and consider the estimated cost impacts alongside evidence from the effectiveness study on the impact of the continuity of care approach on wider clinical outcomes. Results will be subject to sensitivity analysis.

### Trial management and oversight arrangements

The trial will be co-ordinated by the Trial Management Group (TMG) which comprises the Co-ordinating Centre staff, co-applicants and site principal investigator (PI). The Co-ordinating Centre at King’s College London will take responsibility for all aspects of the study including ethical, regulatory, study conduction, data quality and management and publication strategy. The trial manager and designated members of the study team will manage and monitor data, and report to the chief investigator (CI). A delegation list will be prepared for the local site, detailing the delegated responsibilities of each member of staff working on the trial. The local PI will have overall responsibility for the study at the site. A Trial Steering Committee (TSC), composed of independent members (i.e. a consultant obstetrician and gynaecologist; a reader in midwifery/consultant midwife; a professor in paediatrics and neonatal medicine), has also been established to oversee and review the progress of the trial every 6 months. Since this is not a Clinical Trial of an Investigational Medicinal Product, no formal data monitoring committee or interim analysis are required to oversee the safety of subjects in the trial. The TSC will take overall responsibility for the conduct of the trial.

Plans are in place for detecting, evaluating and reporting serious adverse events including safety and progress reporting. All study staff will have evidence of current and ongoing Good Clinical Practice training, maintain participant confidentiality and comply with the requirements of the GDPR and Data Protection Act 1998 with regards to the collection, storage, processing and disclosure of personal information. Protocol amendments, deviations and violations must be reported, reviewed and approved with the CI and appropriate governance and regulatory authorities (if applicable). If a potential serious breach is identified by the CI, PIs or delegates, the sponsor must be notified within 24 h to assess the impact of the breach on the scientific value of the trial and take the appropriate action. The sponsor is responsible for ensuring that proper provision has been made for insurance or indemnity to cover their liability of the CI and staff.

### Patient and Public Involvement (PPI)

Patients and the public were formally involved in the development of the research question and outcome measures, conduct of the study and dissemination. The research question was developed with staff in the Department of Public Health and NHS Clinical Commissioning group in the borough hosting the hospital. We used several strategies for PPI including the discussion of the project and outcome measures important to women and partners in workshops with NIHR South East London PPI event, NIHR Maternity Voices Partnership (MVP) Facebook Group, three locally convened focus groups of Lewisham parents of preterm babies (including fathers) and other events with service-user organisation representatives and charities (e.g. National Childbirth Trust; Tommy’s charity). A lay representative (a service user researcher and PPI lead for maternity services) attends the TSC group. The results will also be disseminated to study participants through individual feedback, organisational newsletter and a feedback event organised with our NIHR CLAHRC South London MVP.

## Discussion

The underlying reasons why midwife continuity of care models are associated with a reduction in fetal loss and fewer PTBs are currently unclear [6]. Little is known about the effects of midwife continuity models of care on mothers’ and babies’ health and wellbeing during and beyond the postpartum period; about the extent to which women feel differently or similarly with different models of care; and about how well these models of care are implemented in health services. Challenges to implementation and scale-up include midwives’ reluctance to change working patterns, midwife concerns about burnout and exploitation, managers concerns about cost, organisational disruption and how to manage change, and what populations to target when whole service change is not possible [20].

This will be the first study to provide direct evidence regarding effectiveness-implementation of a midwifery continuity of care model to improve clinical and processes outcomes in pregnant women at high risk of PTB and determine the feasibility of moving to a full-scale multi-centre trial. Two Cochrane reviews [6, 33] emphasised the need for such a research project as evaluation of these continuity models remains a global research priority to improve the quality of care for every woman and every child [34].

## Trial status

Ongoing. Recruitment and randomisation of participants in the pilot trial started on 11 May 2017 and ended on 30 September 2018. Recruitment of participants for qualitative interviews is expected to end on 30 March 2019.

## Abbreviations

CI: Chief investigator
GCP: Good Clinical Practice
MPV: Maternity Voices Partnership
MRC: Medical Research Council
NHS: National Health Service
PI: Principal investigator
POPPIE: Pilot study Of Preterm birth Including women’s Experiences
PPI: Patient and Public Involvement
PTB: Preterm birth
RE-AIM: Reach Effectiveness Adoption Implementation Maintenance
TIDieR: Template for Intervention Description and Replication
TMG: Trial Management Group
TSC: Trial Steering Committee
UK: United Kingdom
WTE: Whole-time Equivalent

## References

[1] Blencowe H, Cousens S, Chou D, Oestergaard M (2013). Born too Soon: the global epidemiology of 15 million preterm births. Reprod Health.

[2] Petrou S, Eddama O, Mangham L (2011). A structured review of the recent literature on the economic consequences of preterm birth. Arch Dis Child Fetal Neonatal.

[3] Vieira ME, Linhares MB (2016). Quality of life of individuals born preterm: a systematic review of assessment approaches. Qual Life Res.

[4] Chang HH, Larson J, Blencowe H, Spong CY, Howson CP, Cairns-Smith S (2013). Preventing preterm births: analysis of trends and potential reductions with interventions in 39 countries with very high human development index. Lancet..

[5] NICE (2015). Preterm Labour and Birth.

[6] Sandall J, Soltani H, Gates S, Shennan A, Devane D (2016). Midwife-led continuity models versus other models of care for childbearing women. Cochrane Database Syst Rev.

[7] Rayment-Jones H, Murrells T, Sandall J (2015). An investigation of the relationship between the caseload model of midwifery for socially disadvantaged women and childbirth outcomes using routine data—a retrospective, observational study. Midwifery..

[8] Honest H, Forbes CA, Duree KH, Norman G, Duffy SB, Tsourapas A (2009). Screening to prevent spontaneous preterm birth: systematic reviews of accuracy and effectiveness literature with economic modelling. Health Technol Assess.

[9] NHS England National Maternity Review. Better births: improving outcomes of maternity services in England—A five year forward view for maternity care. NHS England; 2016.

[10] Abbott DS, Hezelgrave NL, Seed PT, Norman JE, David AL, Bennett PR (2015). Quantitative fetal fibronectin to predict preterm birth in asymptomatic women at high risk. Obstet Gynecol.

[11] Min J, Watson H, Hezelgrave N, Seed P, Shennan A (2016). Ability of a preterm surveillance clinic to triage risk of preterm birth: a prospective cohort study. Ultrasound Obstet Gynecol.

[12] Hezelgrave NL, Shennan AH (2016). Quantitative fetal fibronectin to predict spontaneous preterm birth: a review. Womens Health.

[13] Fernandez Turienzo C, Sandall J, Peacock JL (2016). Models of antenatal care to reduce and prevent preterm birth: a systematic review and meta-analysis. BMJ Open.

[14] Hoffmann TC, Glasziou PP, Boutron I, Milne R, Perera R, Moher D (2014). Better reporting of interventions: template for intervention description and replication (TIDieR) Checklist and guide. BMJ..

[15] Moore G, Audrey S, Barker M, Bond L, Bonell C, Cooper C (2014). Process evaluation in complex public health intervention studies: the need for guidance. J Epidemiol Community Health.

[16] Glasgow R (2001). The RE-AIM framework for evaluating interventions: what can it tell us about approaches to chronic illness management?. Patient Educ Couns.

[17] Craig P, Dieppe P, Macintyre S, Michie S, Nazareth I, Petticrew M (2008). Developing and evaluating complex interventions: the new Medical Research Council guidance. BMJ..

[18] Liou Shwu-Ru, Wang Panchalli, Cheng Ching-Yu (2016). Effects of prenatal maternal mental distress on birth outcomes. Women and Birth.

[19] Staneva A, Bogossian F, Pritchard M, Wittkowski A (2015). The effects of maternal depression, anxiety, and perceived stress during pregnancy on preterm birth: a systematic review. Women Birth.

[20] Sandall J, Coxon K, Mackintosh N, Rayment-Jones H, Locock L, Page L (2016). Relationships: the pathway to safe, high-quality maternity care. Report from the Sheila Kitzinger Symposium at Green Templeton College October 2015.

[21] Leap NSJ, Buckland S, Huber U (2010). Journey to confidence: women's experiences of pain in labour and relational continuity. J Midwifery Womens Health.

[22] Hetherington E, Doktorchik C, Premji SS, McDonald SW, Tough SC, Sauve RS (2015). Preterm birth and social support during pregnancy: a systematic review and meta-analysis. Paediatr Perinat Epidemiol.

[23] Curran GM, Bauer M, Mittman B, Pyne JM, Stetler S (2012). Effectiveness-implementation hybrid designs. Medic Car.

[24] Public Health England. Public Health Profiles: London Boroughs. https://fingertips.phe.org.uk/profile/health-profiles. Accessed 28 Apr 2019.

[25] Cordoba I, Schwartz L, Woloshin S, Bae H, Gøtzsche PC (2010). Definition, reporting, and interpretation of composite outcomes in clinical trials: systematic review. BMJ..

[26] Alerdice F, Savage-McGlynn E, Martin C (2013). The Prenatal Distress Questionnaire: an investigation of factor structure in a high risk population. J Reprod Infant Psycho.

[27] Krieger N, Smith K, Najshadham D (2013). Experiences of discrimination: validity and reliability of a self-report measure for population health research on racism and health. Soc Sci Med.

[28] International Consortium for Health Outcomes Measurement (ICHOM) (2016). Pregnancy and Childbirth data collection reference guide.

[29] Hodnett ED, Simmons-Tropea DA (1987). The Labour Agentry Scale: psychometric properties of an instrument measuring control during childbirth. Res Nurs Health.

[30] Taylor A, Atkins R, Kumar R, Adams D, Glover V (2005). A new mother-to-infant bonding scale: links with early maternal mood. Arch Womens Ment Health.

[31] Radwin LE, Cabral HJ (2010). Trust in Nurses Scale: construct validity and internal reliability evaluation. J Adv Nurs.

[32] Baker D, Taylor H, The Alspac Survey Team. The relationship between condition-specific morbidity, social support and material deprivation in pregnancy and early motherhood. Soc Sci Med. 1997;45(9):1325–36. 10.1016/s0277-9536(97)00059-2 9351152

[33] Medley N, Vogel JP, Care A, Alfirevic Z (2018). Interventions during pregnancy to prevent preterm birth: an overview of Cochrane systematic reviews (Review). Cochrane Database Syst Rev.

[34] Powell Kennedy H, Yoshida S, Costello A, Declercq E, Dias M (2016). Asking different questions: research priorities to improve the quality of care for every woman, every child. Lancet..

